# Food Insecurity, Diet and Health Outcomes in Pediatric Inflammatory Bowel Disease: A Pilot Study

**DOI:** 10.3390/nu17172730

**Published:** 2025-08-23

**Authors:** Nicole Zeky, Alysse Baudier, Colleen Leblanc, Elizabeth McDonough, Sarah A. Dumas, Dedrick Moulton

**Affiliations:** 1 Cincinnati Children’s Hospital Medical Center, Cincinnati, OH 45229, USA; 2 Division of Pediatric Gastroenterology, Cincinnati, OH 45229, USA; 3 Department of Pediatrics, University of Cincinnati College of Medicine, Cincinnati, OH 45229, USA; 4 Manning Family Children’s, New Orleans, LA 70118, USA; 5 Division of Pediatric Gastroenterology, Louisiana State University-Health Science Center, New Orleans, LA 70118, USA; 6 Department of Pediatrics, Louisiana State University-Health Science Center, New Orleans, LA 70118, USA

**Keywords:** pediatric, inflammatory bowel disease, food insecurity

## Abstract

Background/Objectives: Food insecurity (FI) is a well-defined factor in pediatric health outcomes and has been associated with lower diet quality. While poor diet quality has been linked to the rising prevalence of inflammatory bowel disease (IBD), little is known about the impact of FI on pediatric IBD. This pilot study explores the feasibility and potential impact of FI on dietary intake and clinical outcomes in children with newly diagnosed IBD. Methods: This pilot study included newly diagnosed IBD patients aged 5 to 18. FI screening was completed using the USDA 6-item and AAP 2-item screeners at diagnosis and 6 months. Dietary intake was classified according to their degree of processing (NOVA classification). Clinical data, anthropometrics, and healthcare utilization were collected over 6 months. Results: Among 20 patients, FI was identified in 40% of families. Food-insecure patients had significantly lower weight and BMI z-scores at diagnosis compared to food-secure peers (*p* = 0.002 and *p* = 0.0013, respectively). Food-insecure patients consumed more ultra-processed foods (UPFs, 70.6% vs. 66.7%, *p* = 0.473). However, most patients consumed diets high in ultra-processed foods. FI status was dynamic over the study period. Hospitalizations were more frequent among food-insecure patients. Conclusions: FI is common in pediatric IBD and associated with poorer nutritional status. FI was associated with higher consumption of UPFs, although diet quality was poor among most patients. Future studies should validate these findings in large cohorts and evaluate longitudinal interventions.

## 1. Introduction

Inflammatory bowel disease (IBD), encompassing Crohn’s disease (CD) and ulcerative colitis (UC), is a chronic inflammatory condition primarily affecting the gastrointestinal tract [1]. Both genetic and environmental factors (e.g., exposure to infections, diet, antibiotics) are thought to influence the risk of developing IBD by affecting or impacting the body’s immune response [2,3,4]. Of these, diet has garnered increasing attention, with lower overall diet quality and greater intake of ultra-processed foods (UPFs) being associated with an increased prevalence of IBD, specifically CD [3].

Food insecurity (FI), defined as a lack of consistent access to enough food to live an active and healthy life, affects approximately 11 million US children [5,6]. FI has been associated with a range of adverse health outcomes in children, including emergency department (ED) utilization, developmental delays and heightened risk of mental health disorders [7,8,9,10]. While the burden of FI in pediatric chronic disease populations is not well characterized, existing studies report FI prevalence rates of 17% in cystic fibrosis, 19% in type 1 diabetes mellitus and up to 29% in asthma [11,12,13]. In addition to its health impacts, FI has been linked to lower diet quality, including decreased consumption of fruits and vegetables and increased consumption of UPFs [14,15,16,17,18,19].

Despite the known associations between FI, dietary patterns and clinical outcomes, there remains a critical gap in our understanding of how FI affects pediatric IBD. To address this gap, in this pilot study, we aimed to determine the prevalence of FI among a newly diagnosed cohort of patients at our center. Our secondary aim was to understand the feasibility and potential differences in dietary patterns and clinical outcomes that might be related to FI. We hypothesized that food-insecure patients would exhibit lower diet quality, higher consumption of UPFs, and worse clinical outcomes compared to their food-secure counterparts.

## 2. Materials and Methods

### 2.1. Study Design and Participants

This single-center, pilot cohort study included pediatric patients (ages 5–18 years) newly diagnosed with IBD. Patients were eligible if diagnosed between October 2021 and August 2022. Diagnosis was confirmed through manual review of endoscopic, histologic, and imaging findings. We conducted a directed acyclic graph (DAG) to better understand the association between FI, diet and clinical outcomes. Comprehensive demographic and clinical data were abstracted from the Electronic Medical Record (EMR) from diagnosis through the first six months. Clinical data included disease phenotype, laboratory values and medication use. Additionally, the patient’s median household income was determined by census tract where the patient resided [20]. Caregivers were queried about transportation access, including primary mode of travel to clinic, use of personal versus shared vehicles, number of household vehicles, and bus transfers when applicable. The study was approved by our Institutional Review Board.

### 2.2. Food Insecurity Assessment

All patients over 12 years of age and their parents were screened for food insecurity with both the United States Department of Agriculture’s (USDA) 6-question screening tool and the American Academy of Pediatrics (AAP) 2-question screening tool at both enrollment and study conclusion since FI can change over time [21,22]. Using two questionnaires increased the sensitivity of the screening. Participants were considered food insecure if their questionnaires were positive at either time point from either the patient or the parent. Two different questionnaires were used to increase the sensitivity of our screening process. Parent and patient screens were both used if the patient was aged 12 years or older at enrollment, and for patients younger than 12 years, only the parent screener was used. A positive screen was defined as ≥2 affirmative responses using the USDA screening tool or ≥1 affirmative response using the AAP questionnaire. Additionally, parents were asked if they received Supplemental Nutrition Assistance Program (SNAP) benefits.

### 2.3. Dietary Assessment

Dietary intake was assessed at diagnosis, three months and six months. At each time point, participants were instructed to complete a diet journal for one week. If they were non-adherent to this, then a 24 h diet recall was obtained around each time frame. Each item was classified according to its degree of processing according to the NOVA classification: group 1 (unprocessed or minimally processed), group 2 (processed culinary ingredients), group 3 (processed foods), and group 4 (ultra-processed foods, UPFs) [23]. Diet histories were collected and analyzed by two members of the research team, including a registered dietitian, to ensure consistency and accuracy (AB, NZ).

### 2.4. Clinical and Laboratory Measures

Demographic information (age at diagnosis, sex, race, ethnicity, insurance) and anthropometrics (height z-score, weight z-score, and BMI z-score) were collected at diagnosis, three and six months. Lastly, we collected serum inflammation markers, including C-reactive protein (CRP), erythrocyte sedimentation rate (ESR), albumin, platelet count, hemoglobin and hematocrit levels, at the same time points. Disease severity at diagnosis was also determined and classified as mild, moderate or severe. Mild and moderate disease classification was determined based on endoscopy scores. Severe disease was defined as either (1) an endoscopy score at diagnosis indicating severe disease (SES-CD > 15, MAYO > 3), or (2) stricturing, penetrating, or perianal disease in CD patients. Endoscopy scores were based on the Simple Endoscopic Score for Crohn’s Disease (SES-CD) and the Mayo Endoscopy Score for patients with UC [24,25]. Scoring was performed by members of the research team at the time of data collection and not completed at the time of initial endoscopy. Endoscopy scores were graded by three members of the research team independently (initially scored by NZ, then CL and EM). Healthcare utilization was also collected through the six months and included the number of extra clinic visits. These were defined as extra visits that were added on for patients who were experiencing changes in disease activity. In our practice, patients newly diagnosed with IBD are seen every three months, and, therefore, these visits were added on outside of this expected schedule. Emergency department (ED) visits and hospitalizations were also collected. Lastly, IBD-related surgeries were gathered throughout the study period.

### 2.5. Statistical Analyses

Descriptive statistics were used to summarize baseline characteristics. Continuous variables were reported as mean (±standard deviation) or median (25th and 75th percentiles). Group comparisons of categorical proportions across grouping levels were carried out using Fisher’s Exact Test. Group comparisons for continuous and ordinal variables were performed using the Wilcoxon Rank Sum test. SAS Version 9.4 was used to carry out all comparison tests. This was designed as a pilot study to understand feasibility. These data were collected in order to inform future work so they were not powered for significance testing. All *p*-values are exploratory.

## 3. Results

### 3.1. Patient Characteristics

Twenty newly diagnosed pediatric patients with inflammatory bowel disease (IBD), which included 95% with Crohn’s disease, were enrolled. Most patients (80%) had severe disease at diagnosis (Table 1). In this cohort, all patients started infliximab as their maintenance medication after diagnosis. Overall, eight (40%) households were food insecure based on screening.

### 3.2. Demographic and Clinical Characteristics of Food-Insecure Patients

Among patients who were food insecure, half were white, and most (62.5%) had public insurance (Table 1). The proportion of patients with severe disease was similar between food-secure and -insecure groups (83.3% vs. 75.0%, *p* = 0.901). Despite this, the median C-reactive protein (CRP) value was higher for food-secure patients compared to food insecure (3.85 [2.55, 5.55] vs. 1.25 [0.55, 2.6], *p* = 0.041). No other demographic or clinical differences at diagnosis reached statistical significance between the groups.

**Figure 1:**
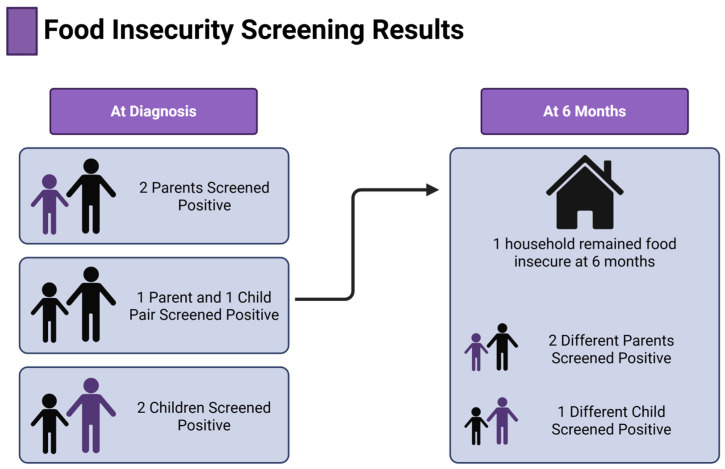
Results of food insecurity screening at diagnosis and at 6 months highlight the dynamic nature of food insecurity.

Screening for food insecurity (FI) varied over time. At enrollment, four households screened positive for FI (two parent and two child responses from separate households). One additional household screened positive based on results from one parent–child pairing. At six months, this same family remained positive for FI. Three new households were identified as food insecure at 6 months, highlighting the dynamic nature of FI status over time (Figure 1).

### 3.3. Dietary Patterns

**Figure 2:**
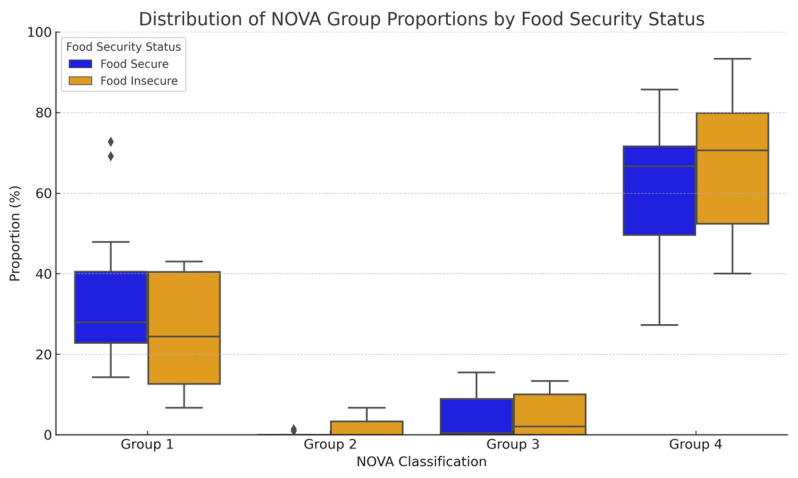
Comparison of box plots of the proportions of foods based on NOVA classification. There were no significant differences between groups based on food security status (group 1 *p* = 0.440, group 2 *p* = 0.205, group 3 *p* = 0.6560, group 4 *p* = 0.473). [Group 1: unprocessed or minimally process foods; group 2: processed culinary ingredients; group 3: processed foods; group 4: ultra-processed foods (UPFs)] [note: visualizations are descriptive and *p*-values are exploratory]. [Note: diamonds represent outliers within the data set].

**Figure 3:**
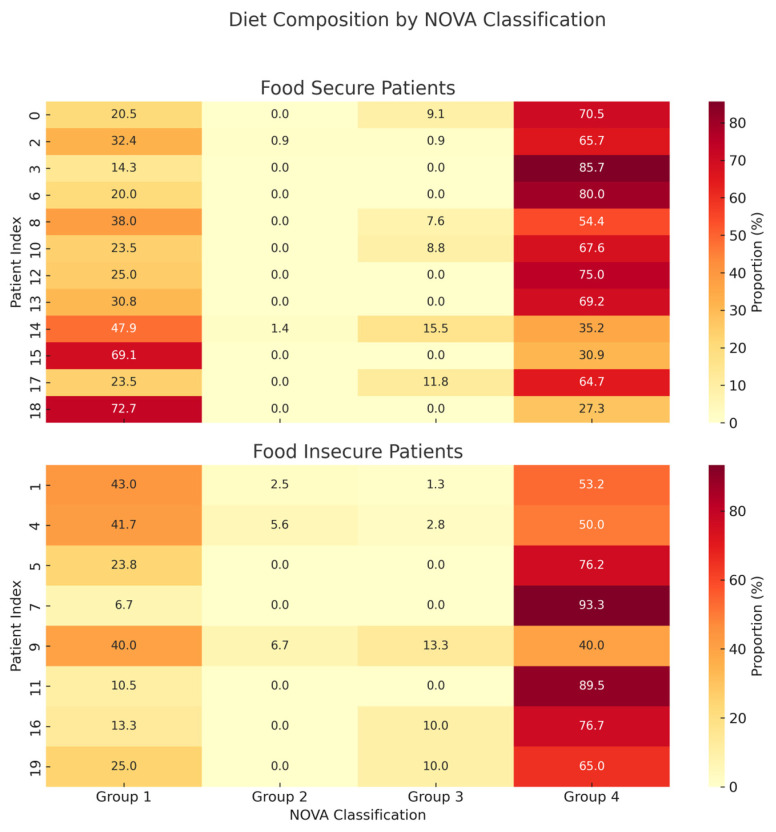
Heatmap showing the dietary pattern of each patient. Most patients consumed a large proportion of their diet as ultra-processed foods (UPFs, NOVA group 4 foods). Although there were no statistically significant differences between the two groups as a whole, some food-secure patients consumed more unprocessed (NOVA group 1) than ultra-processed (NOVA group 4).

Most patients (80%) consumed diets in which over half of the total intake was composed of ultra-processed foods (UPFs). Although not statistically significant, patients in the food-insecure group consumed more UPFs than food-secure patients (70.6 [52.4, 79.9] vs. 66.7 [49.6, 71.6], *p* = 0.473) (Figure 2). Moreover, the food-insecure group consumed lower proportions of group 1, unprocessed foods, compared to the food-secure group (24.4 [12.6, 40.4] vs. 27.9 [22.8, 40.5], *p* = 0.440). UPF consumption varied widely across individuals (Figure 3), ranging from 27.3% to 85.7% among food-secure and from 40.0% to 93.3% among food-insecure patients. Notably, only two participants consumed more than 50% of their diet from unprocessed foods, both of whom were food secure.

### 3.4. Anthropometric Trends and Healthcare Utilization

**Figure 4:**
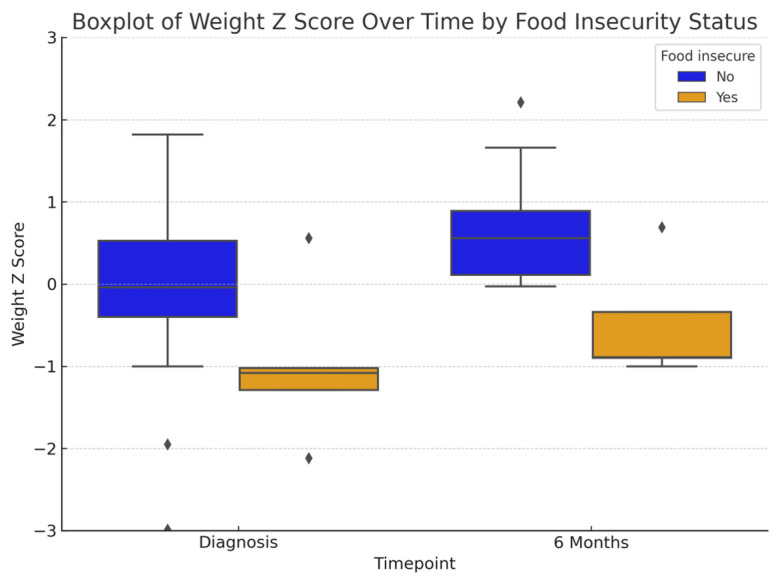
Comparison of box plots with weight z-score changes between both groups at different time points [note: visualizations are descriptive; diamonds represent outliers].

**Figure 5:**
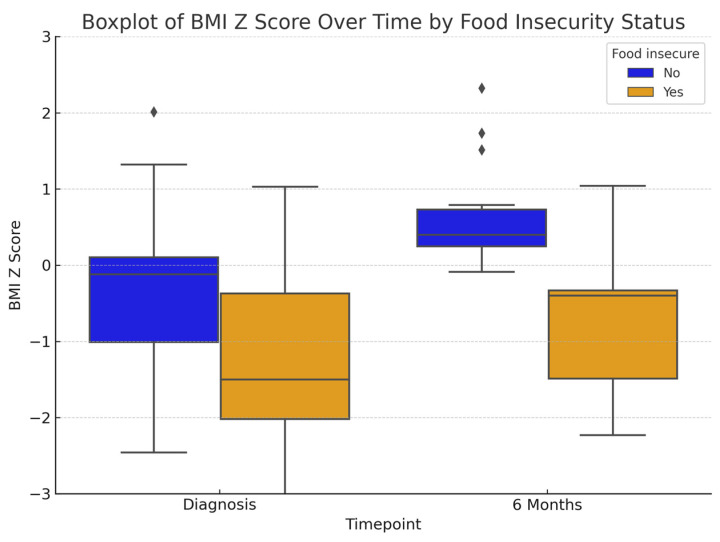
Comparison of box plots with BMI z-score changes between both groups at different time points [note: visualizations are descriptive; diamonds represent outliers].

At diagnosis, food-insecure patients had significantly lower weight z-scores (−0.99 ± 0.97 vs. −0.14 ± 1.21, *p* = 0.002) and BMI z-scores (−1.31 ± 1.78 vs. −0.42 ± 1.35, *p* = 0.0013) compared to those that were food-secure (Table 2, Figure 4 and Figure 5) patients. By six months, weight, height or BMI z-scores were not statistically different between groups; however, food-insecure patients continued to have lower mean values across all anthropometric measures, suggesting persistent nutritional disparities.

Healthcare utilization was generally low. Rates of additional clinic visits, emergency department (ED) visits, and surgeries were similar between groups, but hospitalizations were more frequent among food-insecure patients during the six-month follow-up (Table 2).

## 4. Discussion

In this pilot study of newly diagnosed pediatric patients with IBD, we observed a high prevalence of food insecurity (FI), affecting 40% of families—nearly four-times the national average for households with children [5,6]. This is the first study to document FI prevalence and its association with dietary intake and in pediatric IBD. Food-insecure patients presented with significantly lower weight z-scores, BMI z-scores, but higher consumption of UPFs. By the end of this study, no significant differences were observed, suggesting that optimized medical therapy may have mitigated initial disparities and enabled patients to achieve comparable outcomes. Although FI has been associated with lower diet quality, it may also influence the quantity of food consumed [15,26,27,28,29]. Notably, most patients, regardless of food security status, consumed a diet high in UPFs, indicating poor diet quality across the cohort. These findings suggest that dietary intake is influenced by a complex interplay of factors beyond FI, many of which have yet to be fully elucidated.

Food insecurity is disproportionately prevalent in the southern United States, particularly among single-parent households and families that identify as Black or Hispanic [6]. All patients in our cohort resided in Louisiana or Mississippi, which rank among the highest in poverty, single-parent households, and underrepresented racial and ethnic groups likely reflecting these disparities [30]. Our prior work also showed that food deserts are highly prevalent in this area, further compounding the risk of FI [31]. Although FI was not associated with significant differences in disease severity or clinical remission, its presence alone warrants clinical attention. FI may act as a downstream social determinant of health (SDOH) that interacts with other factors, including income, transportation access, and health literacy, to shape dietary behaviors and health outcomes in children with IBD. Importantly, FI status fluctuated during the study period, with some families screening positive at one time point and not the other. This dynamic nature underscores the need for routine, repeated FI screening in pediatric IBD care, where early identification may inform targeted nutritional and social interventions [32,33,34].

Prior studies have demonstrated that FI is associated with poorer-quality diets and greater reliance on UPFs [19,26,27,35]. In our cohort, UPF intake was high among all patients, regardless of food security status. This suggests that factors beyond FI, such as sociodemographic context, cultural influences, and perceptions about food triggers, may strongly shape dietary patterns [17,36,37]. In adult IBD, more than half of patients report altering their diets post-diagnosis, often without professional guidance, with 74% believing diet plays an important role in disease and only 15% thought it could be a trigger for their IBD [38]. Pediatric data are limited on this subject, but one study reported that 48% of patients and 60% of parents thought that eating certain foods can trigger disease flares [39]. Despite beliefs, most families did report making dietary changes after their child’s IBD diagnosis, often eliminating dairy and vegetables [39]. Our findings, in the context of the literature, highlight that dietary behavior in pediatric IBD is complex, multifactorial and not solely determined by food security status, underscoring the need for ongoing research in this area of pediatric IBD care.

This common practice is one that goes against what is understood about the role diet plays in IBD. A Mediterranean Diet, naturally high in NOVA 1, unprocessed foods, has been associated with improvements in clinical disease activity scores and inflammatory markers in pediatric IBD patients [40]. Mechanistically, the Mediterranean Diet is thought to alter the gut microbiome’s diversity, leading to anti-inflammatory effects, although work in this area remains ongoing [41,42,43]. Similarly, greater consumption of unprocessed NOVA 1 foods has been linked to lower risk of developing Crohn’s disease [44]. The near-universal reliance on UPFs in our cohort represents a missed opportunity to leverage diet as an adjunctive tool in IBD care. However, we recognize that Crohn’s disease (CD) and ulcerative colitis (UC) are two distinct entities, each with different presentations, phenotypes, and pathophysiology. Notably, our study included mostly CD patients, where the role of dietary changes has been better established including the impact of diet on the mucosal integrity—factors implicated in the pathogenesis of CD. These findings underscore the importance of early nutritional counseling and structured dietary interventions as part of comprehensive, multidisciplinary IBD management, specifically differences targeted toward those with CD versus UC.

**Figure 6:**
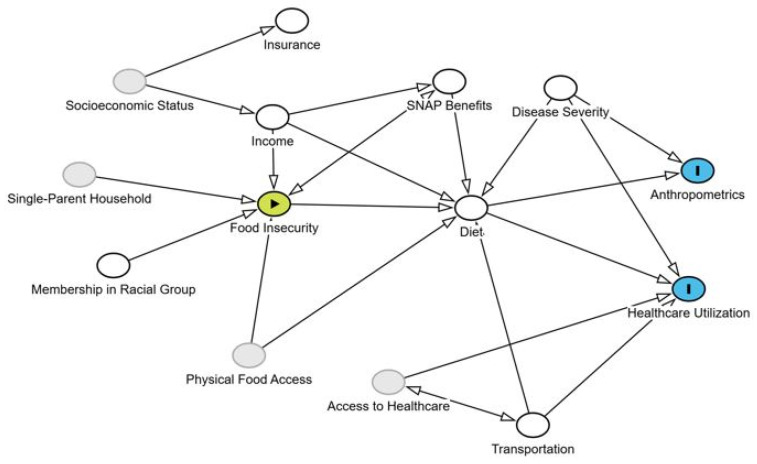
Directed acyclic graph (DAG) depicting potential pathways between food insecurity and clinical outcomes (blue) in inflammatory bowel disease. This diagram illustrates hypothesized relationships among social, economic, and clinical factors that may influence outcomes in patients. White ovals represent those variables that were collected in this study. Gray ovals represent other variables that may influence this association but were unobserved in this study. Food insecurity (yellow) is influenced by several other social determinants of health. This framework can guide variable selection and helps to identify potential confounding factors for future analyses.

While healthcare utilization overall was low, food-insecure patients experienced a higher rate of healthcare utilization over the six-month follow-up period. In adult IBD, FI was associated with greater healthcare utilization, as demonstrated by increased IBD-related hospitalizations, surgeries and ED visits [45,46]. Food insecurity may serve as a proxy for other unmeasured barriers to care including transportation and access to healthcare. Damas et al. highlighted that a higher social barriers score, which included various SDOH including FI, was associated with worse clinical outcomes, suggesting the complexity of these factors being considered [47]. Additionally, food-insecure patients in our study exhibited significantly lower weight and BMI z-scores at diagnosis. The persistence of lower anthropometrics indices among food-insecure patients suggests potential nutritional disparities that need to be explored further. To better understand the complex, multi-level relationships between FI, diet, and IBD outcomes, we developed a directed acyclic graph (DAG) illustrating potential causal pathways (Figure 6). Structural and socioeconomic factors intersect with clinical care and household behaviors, highlighting the need for longitudinal and systems-level research to further elucidate these interactions. While our pilot study measured several of these variables, future work should incorporate additional factors to better capture upstream determinants.

This study has several limitations. It was conducted at a single center with a small sample size, limiting generalizability and power to detect certain group differences. Our intent was to assess feasibility and generate preliminary data that we hope will inform future work. Another limitation was the limited follow-up period, which may not have allowed other clinical outcomes related to food insecurity to emerge. Dietary intake was self-reported and may be subject to recall bias, although dietitian review aimed to minimize this. Additionally, although all patients received infliximab, we were unable to fully account for adherence or pharmacokinetics that might affect treatment response. Despite these limitations, our findings offer novel insights into dietary intake and food insecurity in pediatric IBD. We recognize the potential for implicit bias in study design and the need for replication in larger, more diverse populations. Future studies should aim to include a diverse population of patients across several centers to enhance the generalizability of the findings demonstrated here. Moreover, future studies can include incorporating fecal biomarkers or microbiome profiles to better provide mechanistic insights into the role food insecurity might have on diet quality and gut health in pediatric IBD.

## 5. Conclusions

Food insecurity is common in pediatric IBD and should be routinely screened for alongside other SDOH as part of comprehensive, multidisciplinary care. In this pilot cohort, all patients consumed a high proportion of UPFs; these findings underscore the gap in nutrition-based patient education. Future research should focus on larger studies to validate these results and better understand the influences of dietary intake. Future studies should aim to better understand the various components that drive oral intake and how to ultimately improve diet quality, especially limiting the consumption of UPFs. Targeted nutrition-based interventions addressing diet quality should be integrated into multidisciplinary IBD care to improve health equity and clinical outcomes.

## Figures and Tables

**Table 1:** Patient characteristics by food security status.

	Food Secure (*n* = 12)	Food Insecure (*n* = 8)	*p*-Value
Sociodemographic Information			
Age	15.0 (13.8, 16.2)	14.0 (12.8, 16.2)	0.755
Male	4 (33.33%)	2 (25%)	1.000
Race Black/Other White	6 (50.0%) 6 (50.0%)	4 (50.0%) 4 (50.0%)	1.000
Ethnicity Non-Hispanic	12 (100.0%)	8 (100.0%)	1.000
Insurance Private Public	6 (50.0%) 6 (50.0%)	3 (37.5%) 5 (62.5%)	0.670
Receives SNAP Benefits	5 (41.7%)	5 (62.5%)	0.6499
Transportation Own Car Someone Else’s Car Bus	10 (83%) 0 2 (17%)	7 (87.5%) 1 (12.5%) 0	0.2413
Median Household Income	63,064 (44,073, 80,159)	48,193 (39,207, 54,803)	0.1813
IBD Type and Characteristics			
Crohn’s Disease Ulcerative Colitis *	11 (91.7%) 1 (8.3%)	8 (100.0%) 0 (0.0%)	1.000
Disease Severity at Diagnosis Mild Moderate Severe	1 (8.3%) 1 (8.3%) 10 (83.3%)	1 (12.5%) 1 (12.5%) 6 (75.0%)	0.901
Disease Location for Crohn’s Disease Patients			
L1 (ileal) L2 (colonic) L3 (ileocolonic)	4 (36.4%) 1 (9.1%) 6 (54.5%)	1 (12.5%) 3 (37.5%) 4 (50%)	0.2471
Any L4ab/L4b (upper tract)	5 (45.5%)	2 (25%)	0.6424
Inflammatory Phenotype	8 (72.7%)	4 (50%)	0.2660
Perianal Disease	0	2 (25%)	0.1474
Laboratory Values at Baseline			
CRP	3.85 (2.55, 5.55)	1.25 (0.55, 2.6)	0.041
ESR	33 (28, 45)	32 (21.5, 68.5)	0.918
Albumin	3.2 (3.0, 3.8)	3.9 (2.7, 4.0)	0.352
Hemoglobin	12.0 (10.7, 12.5)	9.6 (7.9, 12.2)	0.105
Vitamin D	23.1 (15.6, 31.3)	20.9 (15.8, 26.2)	0.473
Vitamin B12	511.5 (378.5, 685.2)	629.0 (501.5, 764.5)	0.482
Folate	11.6 (9.2, 18.8)	14.2 (11.2, 19.2)	0.687
Zinc	80.0 (68.0, 97.5)	79.0 (67.8, 96.0)	0.855
Ferritin	21.9 (14.6, 61.2)	28.3 (23.5, 34.5)	1.000
TIBC	285.0 (234.7, 359.4)	240.8 (189.5, 282.1)	0.216

Values expressed as counts and proportions or median (interquartile range, 25th centile, 75th centile. Abbreviations: IBD: inflammatory bowel disease; SNAP: Supplemental Nutrition Assistance Program; CRP: C-reactive protein; ESR: Erythrocyte Sedimentation Rate; TIBC: Total Iron Binding Capacity. * One patient with UC had pancolitis (E4) for disease phenotype.

**Table 2:** Outcomes through the first 6 months.

	Food Secure (*n* = 12)	Food Insecure (*n* = 8)	*p*-Value
Anthropometrics at Diagnosis			
Height z-score	0.28 ± 0.89	−0.21 ± 0.95	0.1005
Weight z-score	−0.14 ± 1.21	−0.99 ± 0.97	0.0020
BMI z-score	−0.42 ± 1.35	−1.31 ± 1.78	0.0013
Anthropometrics at 6 Months			
Height z-score	0.14 ± 0.85	−0.12 ± 0.91	0.4603
weight z-score	0.68 ± 0.67	−0.49 ± 0.71	0.0841
BMI z-score	0.67 ± 0.68	−0.68 ± 1.25	0.1677
Healthcare Utilization Through 6 Months			
Extra Clinic Visits ^≠^	0 (0,1)	2 (0, 2.25)	0.1259
ED Visits	0 (0, 2.5)	1 (0,1)	0.0890
Hospitalizations	0 (0, 0)	0 (0,1)	0.0292
Surgeries	0 (0, 0)	0 (0, 0)	0.2616

Values expressed as proportions, median mean ± standard deviation. Abbreviations: BMI: Body Mass Index, ED: emergency department. ≠ denotes clinic visits that needed to be added on to see a patient who was experiencing more symptoms. Typical clinic visits were completed every 3 months.

## Data Availability

The original contributions presented in this study are included in the article. Further inquiries can be directed to the corresponding author(s).
